# Evaluation of different detector types in measurement of ATP bioluminescence compared to colony counting method for measuring bacterial burden of hospital surfaces

**DOI:** 10.1371/journal.pone.0221665

**Published:** 2019-09-06

**Authors:** Huiqiong Xu, Jiansheng Liang, Yimei Wang, Bin Wang, Tianbao Zhang, Xiaoli Liu, Lin Gong

**Affiliations:** 1 Department of Disinfection and Pest Control, Wuhan Centers for Disease Prevention and Control, Wuhan, Hubei, China; 2 Department of Infectious Disease Prevention, Hubei Center for Disease Control and Prevention, Wuhan, Hubei, China; University of Maryland School of Medicine, UNITED STATES

## Abstract

The ATP bioluminescence method has been increasingly employed as a rapid, on-site detection method in nosocomial infections control. In this study, we used a paired design of monitoring methods, the colony counting method (C) and the ATP bioluminescence method, to evaluate environmental surfaces after disinfection. The ATP bioluminescence method included three detector types (B, P, and N). Every surface after disinfection was performed by combining two types of monitoring methods or detectors. There was no statistically significant difference in theATP content per surface siteamong samples from intensive care units (ICUs)and internal medicine wards using B (*p* = 0.435) and P (*p* = 0.260). According to the Spearman’s rank correlation coefficients, with the exception of the correlation between the ATP content values detected by B and P, the correlation between the values generated by the remaining methods/detectors was weak or lacking, whereasthe differences between the detectors were statistically significant. Therefore, there are differences between the ATP bioluminescence method and the colony counting method, also between different detectors.

## Introduction

Effective environmental surface disinfection is essential for the prevention and control of nosocomial infections, i.e., the cleaning quality and disinfection effect are critical for ensuring the safety of patients and medical staff and reducing the risk of nosocomial infections [1–3]. The microbial culture (colony counting method) is the traditional and most common technique to evaluate cleanliness and the disinfection effect. However, it is time-consuming, and in the recent decade, most medical institutions, CDCs, and healthcare supervising organizations in China have begun to use the ATP bioluminescence method to validate the surface disinfection of objects on-site widely. Because of its simplicity and rapidity, the ATP bioluminescence method can solve the problems associated with the delayed results from the colony counting method and monitor the cleanliness of the ward environment according to the actual needs. Its use achieves “all-staff, full-time, comprehensive” monitoring, ensuring that medical sites maintain a high standard of cleanliness over a long time, which strengthens hospital cleanliness and reduces the risk of infection [4].

The ATP bioluminescence method can detect the ATP content in a sample bymonitoring the biological luminescence reaction of a luciferase assay with a luminometer to detect the presence of microorganisms or other organic residues indirectly [5]. Since ATP bioluminescence technology was first described in 1963 by McElroy [6], the method has been introduced into food hygiene [7], nosocomial infections control, and other fields. Our research team has used *Staphylococcus aureus*, *Escherichia coli*, and *Candida albicans* as representative test microorganisms for Gram-positive bacteria, Gram-negative bacteria, and fungi. We analyzed the data by using the correlation coefficient (*r*) to describe the standard validity and the intra-class correlation coefficient (*ICC*) for reliability evaluation. The results show that the ATP bioluminescence method hasgood accuracy and reliability[8]. Some studies [9–11] demonstrateda good correlation between the ATP bioluminescence method and the colony counting method. Therefore, the ATP bioluminescence methodis widely used in nosocomial infection control as an on-site rapid detection method for environmental sanitation disinfection, medical staff hand hygiene, and medical device cleaning effect evaluation [12–15].

However, due to the absence of uniform evaluation standards, most medical institutions rely on the reference values provided by the manufacturer of the testing detectors. In China, the relevant manufacturers recommend that the desktop ATP bioluminescence detector has a threshold RLU≤2,000 [16] as the qualifying criterion for medical device cleaning, whereas the portable ATP bioluminescence detector standards vary between RLU<30[17] and RLU≤500 [18]. This generates uncertainties in result evaluation and creates difficulties for the majority of the medical staff involved in real-time monitoring work. Therefore, our study aimed to compare the ATP bioluminescence method with the colony counting method and the ATP bioluminescence method, also compare differentATP bioluminescencedetectortypes, for monitoring the disinfection effect of environmental surfaces. The RLU values measured by different detectors always vary due to differences in the photometric capability between photomultiplier tubes or in reagent sensitivity. However, the presence of ATP in microorganism is certain, and the difference between the detector types should not be statistically significant after converting the RLU value into ATP content (mol). For this reason, we used standard curves to convert the RLU measurements into ATP content and to allow direct comparison between the readings from two different detector types. Our study wouldevaluate the reproducibility of results from ATP bioluminescence across different detector types and their comparison to colony count methods of assessing bacterial burden of hospital surfaces. And we use the Hygienic Standard for Disinfection in Hospitals(GB15982-2012) [19] to perform the colony count methods, which requiresthe surface of type II environment (including intensive care units) to have a CFU ≤5 and type III environment (including internal medicine wards) a CFU ≤10 after disinfection.

## Methods

### Paired design

There were two principal methods, the colony counting method (C) and the ATP bioluminescence method; the latter was performed by three detector types (B, P, and N). In this study, each sampling was performed according to the two methods or detectors described above (paired design). Specifically, the two methods/detectors were used to sample different points on the same surface, and acquired equal areas. That was, there were 6 groups of pairs, respectively C vs B, C vs P, C vs N, B vs P, B vs N, P vs N.

### Sampling sites

We performed environmental surface tests in 22 medical institutions, including 12 tertiary hospitals, 7 secondary hospitals, and 3 community health service centers. Among the selected hospitals, 13 were comprehensive hospitals, and 6 were specialized hospitals. Intensive care units (ICUs) and internal medicine wards were used as representative sites for type II and type III environments which were defined inGB15982-2012 [19], respectively. The samples were collected fromtreatment vehicles, treatment tables, bedside cabinets,doorknobs, etc. In total, we gathered 670 samples, 303 from ICUs and 367 from internal medicine wards. The study was carried out in 2017 and 2018.

### Disinfection and sampling method

The environmental surfaces were disinfected by chlorine with a disinfectant concentration of 500–1000 mg/L and sampled after the surfaces had dried. We sampled using the swabs in the ATP Surface Test or cotton swabs soaked in neutralizer (0.1% sodium thiosulfate). Surface samples in this study were collected by wiping a 100cm^2^ area with a sterilized specification plate (5cm×5cm). If the total surface at a site was smaller than 200cm^2^, each method or detector was used on half of the area.

### ATP bioluminescence method

Three ATP bioluminescence detectortypes were selected that are most commonly used in Wuhan, Hubei province:B (BT-112D, Beijing ChuangxinShiji Biochemical Science&Technology Development Co., Ltd., China, Precision: 1.0×10^−18^ mol ATP, RSD<3%), P (SystemSURE Plus, Hygiena, Britain, Precision: 1.0×10^−18^ mol ATP, RSD≤5%), and N (Clean-Trace NGi, 3M, USA, Precision: 1.0×10^−15^ mol ATP, CV≤7.4%). Two ATP surface test stickswere used for the detectorsinvolving a swab and some reagents which were standard quantity, the Clean-Trace^™^ ATP Surface Test (N dedicated, Clean-Trace, 3M, USA) and the UltraSnap^™^ ATP Surface Test (B and P dedicated, Ultrasnap, Hygiena, USA). The samples were shaken 20 times to mix the swab with the reagents after sampling and then analyzed by the detector to measure the RLU value. The results were reported as ATP content (mol) per surfacesite, using a standard curve for data conversion.

To prepare the standard curve, the ATP standard solution (100nmol, BioThema, Sweden) was serially diluted in pure water to obtain the following concentrations:1.0×10^−7^ mol/L, 5.0×10^−8^ mol/L, 1.0×10^−8^ mol/L, 5.0×10^−9^ mol/L, 1.0×10^−9^ mol/L, 5.0×10^−10^ mol/L, 1.0×10^−10^ mol/L, 5.0×10^−11^ mol/L, 1.0×10^−11^ mol/L, 5.0×10^−12^ mol/L, and 1.0×10^−12^ mol/L. Aliquots of10 μL were removed from each dilutionanddropped onto the swabto obtain the RLU value as described above. Three replicates of each concentration were processed and used for calculating the average. One detector of each type were selected, and the curve fitting of the ATP content and RLU was carriedout by linear, quadratic, cubic, power and exponential curves respectively. Then, according to the determination coefficient (*R*^2^), the number of coefficients and the professional meaning, a more appropriate curve was selected. Finally, the standard curve x- and y-axis were log-transformed using the base 10 for the ATP content (10^−17^ mol) and RLU; the graph function was y = ax+b. There was an individual standard curve for each detector. (dx.doi.org/10.17504/protocols.io.49hgz36).

### Colony counting method

The colony counting method is a classical microbiological method. After sampling, the nutritional agar culture medium (Qingdao Hope Bio-technology Co., LTD., China) was used to monitor microbial contamination of each surface. The sample processing was performed according to GB15982-2012 [19]. The total colony count was transformed to CFU per surfacesite, which was calculated by multiplyingthe average colony numbers per dish with the sample dilution factor.

### Statistical analysis

The raw data were transferred to Microsoft Office Excel spreadsheetsto conduct the statistical analysis using SPSS version 16.0 with p<0.05, which wasconsidered statistically significant. The results were analyzed separately according to the different source (ICU and internal medicine wards), and the data analysis was done using a nonparametric test. The Kolmogorov-Smirnov test was used to test the normal distribution. The rank correlation was used to compare CFU and ATP content or ATP contents from different detectors, and the Wilcoxon signed-rank test was used to compare ATP content values between different detectors.

## Results

### Standard curve

In this study, we used 3 B, 3 P, and 6 N. In the standard curve experiment, the detection limit was 1.0×10^−17^ mol ATPfor the desktop detector (B) and 1.0×10^−15^ mol for the portable detectors (P and N). According to the curve fitting of B(1), P(1) and N(1), the *R*^2^ of linear, quadratic, cubic and power curves for each detector are similar (*P*<0.001), but linear curve has less coefficients, is more uncomplicated, and can be explained in a professional sense. In addition, the *R*^2^ of linear curve fitting of B(1) using log-transformed with the base 10 (*R*^2^ = 0.974) is relatively better than the original value (*R*^2^ = 0.942). Therefore, the linear curve is more appropriate, which x- and y-axis are log-transformed using the base 10 for the ATP content (10^−17^ mol) and RLU. The standard curve and linear correlation coefficient (*r)* of each detector are shown in Table 1.

**Table 1:** The standard curve and linear correlation coefficient (*r)* of each detectors.

Detector	Standard curve^a^	*r*	Detector	Standard curve^a^	*r*
B (1)	y = 0.893x+0.990	0.987	N (1)	y = 0.914x-0.785	0.996
B (2)	y = 0.987x+1.378	0.999	N (2)	y = 0.905x-0.761	0.996
B (3)	y = 1.004x+0.862	0.998	N (3)	y = 0.931x-0.852	0.998
P (1)	y = 0.941x-1.714	0.990	N (4)	y = 0.874x-0.409	0.995
P (2)	y = 1.022x-0.241	0.996	N (5)	y = 0.851x-0.356	0.994
P (3)	y = 1.002x-1.992	0.998	N (6)	y = 0.853x-0.388	0.994

^a^ x and y were fitted with the base 10 logarithms of the ATP content (10^−17^ mol) and RLU.

### Distribution of the CFU/ATP content

The Kolmogorov-Smirnov test indicated that the distribution of the CFU counts and ATP content measurements in this study did not have a normal distribution and a *p* value of <0.05. Hence, we used the median and inter-quartile range (*Q*) to describe the central tendency and discrete tendency, respectively (Table 2). In this study, 286 C samples (88.0%) were less than or equal to 10 CFU/surfacesite and 218 (67.1%) C samples were 0 CFU/surfacesite. According to GB15982-2012 [19], 276 C samples (84.9%) met the hygienic standard, which required ICUsto have a CFU ≤5 and internal medicine wards a CFU ≤10. According to the Wilcoxon rank sum test, the CFU/ATP content per surface sitewas lower in ICU samples than in internal medicine ward samples gathered by C and N, with associated *p* values of 0.024 and 0.002, respectively. However, there was no statistically significant difference in the ATP content per surfacesite from ICU and internal medicine ward samples of B and P, with associated *p* values of 0.435 and 0.260, respectively.

**Table 2:** Distribution of CFU and ATP content per surface site after disinfection.

Method/Detector	Source	*n*	*Median*	*Q*	*Minimum*	*Maximum*	*P* of Kolmogorov-Smirnov test
C (CFU)	ICU	155	0.0	0.00~1.00	0.0	95.0	<0.001
	Internal medicine wards	170	0.0	0.00~3.00	0.0	1 900.0	<0.001
B (10^−17^ mol)	ICU	201	436.24	123.32~3280.43	8.78	331 911.00	<0.001
	Internal medicine wards	238	358.67	131.70~2033.11	8.78	98 142.00	<0.001
P (10^−17^ mol)	ICU	182	581.60	99.32~1941.85	50.00	72 200.00	<0.001
	Internal medicine wards	227	678.32	194.29~2995.08	66.29	120 400.00	<0.001
N (10^−17^ mol)	ICU	135	183.42	89.73~328.88	27.22	1 845.60	<0.001
	Internal medicine wards	170	251.04	108.07~558.39	27.22	5 440.99	<0.001

### Correlation between different methods/detectors

According to the Spearman rank correlation coefficients (*r*_*s*_), with the exception of the acceptable correlation between the ATP content values measured by B and P, the correlation between the remaining methods/detectors was weak or lacking (Table 3).

**Table 3:** Correlationbetween different method/detector to determine the CFU and ATP content (per surface site), using the Spearman rank correlation coefficients (*r*_*s*_).

Method/Detector	C (CFU)			B (10^-17^mol)			P (10^−17^ mol)			N (10^−17^ mol)		
	*r*_s_	*P*	*n*	*r*_s_	*P*	*n*	*r*_s_	*P*	*n*	*r*_s_	*P*	*n*
C (CFU)	-	-	-	0.293^a^	0.005^a^	90^a^	0.244^a^	0.029^a^	80^a^	0.313^a^	0.049^a^	40^a^
B (10^−17^ mol)	0.450^b^	<0.001^b^	93^b^	-	-	-	0.904^a^	<0.001^a^	121^a^	0.246^a^	0.062^a^	58^a^
P(10^−17^ mol)	0.485^b^	<0.001^b^	90^b^	0.931^b^	<0.001^b^	139^b^	-	-	-	0.073^a^	0.613^a^	51^a^
N (10^−17^ mol)	0.564^b^	<0.001^b^	40^b^	0.378^b^	<0.001^b^	81^b^	0.466^b^	<0.001^b^	70^b^	-	-	-

^a^ It shows the result from the ICUs.

^b^ It shows the result from the internal medicine wards.

### Comparison between detectors

The Wilcoxon signed-rank test did not find a statistically significant differencein the ATP content of the ICU samples detected by P and N, whereas the other ATP content values detected by the other detector combinations showed statistically significant differences (Table 4).

**Table 4:** Comparison of the ATP content values (10^-17^mol) measured using different detectors, using the Wilcoxon signed-rank test.

Paired Detectors	*n*	Source	*Z*	*P*
P&B	121	ICU	-6.512	<0.001
	139	internal medicine wards	-3.951	<0.001
	260	-	-7.475	<0.001
N&B	58	ICU	-3.132	0.002
	81	internal medicine wards	-3.515	<0.001
	139	-	-4.726	<0.001
P&N	51	ICU	-1.790	0.073
	70	internal medicine wards	-2.917	0.004
	121	-	-3.483	<0.001

## Discussion

Contamination of hospital surfaces plays an important role in the transmission and diffusion of several pathogens, which may infect patients, then contaminate the hands of the medical staff and can be potentially passed on to other patients or contaminate other surfaces. This circulation may lead to the occurrence or even an outbreak of nosocomial infections. Consequently, the methods to monitor hospital environment cleaning are an integral part of infections prevention and control [20]. The currently most widespread methods are visual inspection, microbial culture, fluorescent markers, and ATP bioluminescencedetection, which are recognized by the CDC of the USA as the main methods [21]. To date, the ATP bioluminescence method is increasingly used to evaluate the effect of environmental surface cleaning and disinfection. An independent study [22] evaluating the potential role of the ATP methodin assessing the cleaning practicenoted several limitations in the ATP procedurebut concluded that the method could be potentially used effectively in training and education forenvironmentalservices. Boyce JM [23, 24] reported that monitoring the disinfection effectiveness by the ATP bioluminescence assay could significantly improve the daily cleaning.

In order to make different detectors comparable, we used standard curves to convert the RLU measurements into ATP content, and then compare the ATP content values between different detectors. The results show that, after disinfection, the correlation between the ATP content and the CFU number was weak, and the correlation of the ATP content values among the three detectors was also low(Table 3), which might be related to the low detection rate using microbial cultures. In our study, 88% of the CFU counts were less than or equal to 10 CFU/surface, and 67% had 0 CFU/surface. In 2003, Hattori et al. detected 54 microorganism types, and the ATP content of the Gram-negative bacteria, Gram-positive bacteria, and yeasts ranged from 0.40 to 2.70×10^−18^ mol/CFU (mean = 1.5×10^-18^mol/CFU), from 0.41 to 16.7×10^−18^ mol/CFU (mean = 5.5×10^−18^ mol/CFU), and from 0.714 to 54.6×10^−18^ mol/CFU (mean = 8.00×10^−18^ mol/CFU), respectively [25]. According to this analysis, more than 80% of the ATP content values in our study are approximately 10^−17^ mol, but the detection limits of the detectors used in our study were 10^−17^ mol (B) and 10^−15^ mol (P and N). It is possible that the surface after disinfection is too clean and may not be suitable for testing using the ATP bioluminescence method. Furthermore, the differences between the detector types werestatistically significant (Table 4). The homogeneity of the samples may pose another problem for this method. Uneven distribution within a sample could be caused by the presence of chemicals and other materials, such as the residues of detergents or disinfectants, microfiber products, and manufactured plastics [20, 26]. The ATP detection value may also vary depending on the raw material composition of the detected object [27].

There are several limitations to our study. The ATP bioluminescence method can detect different types of organic material, including bacteria, blood, excretions, human secretions, food, etc. [28]. Thus, to evaluate the disinfection effect more accurately, the surface areas may have to be cleaned before disinfection. But in our study, we did not specifically emphasize the role of cleaning. Therefore, because the ATP bioluminescence method detects not only the ATP of microorganisms, but also organic material, it may be more appropriatelyused for evaluating the cleanliness or as an early warning method for microbial contamination. On the other hand, we did not test the background before disinfection, and all of the experiments were completed after disinfecting surfaces. In the absence of post-disinfection evaluation criteria, our study suggests that there are differences between the ATP bioluminescence method and the colony counting method, also between different detectors. It means that it may be not possible to compare the results of the surfaces after disinfectionevaluated by these detectors with the same criteria, and it may be more convincing to use contrast before and after disinfection. In addition, the relationship and differences between the ATP bioluminescence method and the colony counting method in the on-site bacterial contamination detection also require more data to illustrate. Thus, we will continue to assessthe surface cleanliness of environmental objects, and before-and—after testing of disinfection to expand the data support for using the ATP bioluminescence technique as a rapid on-site test.

## Supporting information

S1 File Raw data. (XLSX)
